# Supplementary material for: Early Growth Response 1 Contributes to Renal IR Injury by Inducing Proximal Tubular Cell Apoptosis
Source: Int J Mol Sci. 2023 Sep 19;24(18):14295. doi: 10.3390/ijms241814295 (PMC10532368; doi:10.3390/ijms241814295)
Supplement: Supplementary file 1 [file ijms-24-14295-s001.zip › ijms-2613205-SI.pdf]

Supplementary Table S1. Antibodies used in this study

Antibodies		Vendor	Catalog No.	Host	Application
Mouse and human	Egr-1	Cell signaling	#4153	Rabbit	1:1000 (WB)<br>1:50 (IHC, IF)
	AQP1	Santa Cruz	sc-25287	Mouse	1:50 (IF)
	Calbindin	Abcam	ab75524	Mouse	1:50 (IF)
	p53	Cell signaling	#2524	Mouse	1:1000 (WB)
	Caspase-3	Cell signaling	#9662	Mouse	1:500 (WB)
	Ly-6B.2	Bio-Rad	MCA771G	Rat	1:200 (IHC)
	pErk1/2	Cell signaling	#4377	Rabbit	1:1000 (WB)
	Erk1/2	Cell signaling	#4696	Mouse	1:1000 (WB)
	p-p38	Cell signaling	#9211	Rabbit	1:1000 (WB)
	p38	Cell signaling	#9212	Rabbit	1:1000 (WB)
	p-JNK	Cell signaling	#9251	Rabbit	1:1000 (WB)
	JNK	Cell signaling	#9252	Rabbit	1:1000 (WB)
	β-actin	Sigma-Aldrich	#A5441-2ML	Mouse	1:10,000 (WB)
	Goat anti- Rabbit IgG-Alexa Fluor 488-Green	Abcam	ab150081	Rabbit	1: 200 (IF)
	Goat anti-Mouse IgG-Alexa Fluor 594-Red	Abcam	ab150116	Mouse	1:200 (IF)

Supplementary Table S2. The primer sequences used for real-time PCR analysis in this study

Gene		Forward primers (5′-3′)	Reverse primers (3′-5′):
Mouse	*Egr-1*	CACCCACCATGGACAACTAC	GGTGCTGCTGCTGCTATTA
	Tnfα	CATATACCTGGGAGGAGTCT	GAGCAATGACTCCAAAGTAG
	Mip-2	AGAGGGTGAGTTGGGAACTA	GCCATCCGACTGCATCTATT
	IL-6	GACTTCCATCCAGTTGCCTTCTTG	GGTATCCTCTGTGAAGTCTCCTCT
	Gapdh	GTGGCAAAGTGGAGATTGTTG	TTGACTGTGCCGTTGAATTTG
Human	*Egr-1*	CGCAAGAGGCATACCAAGAT	GTAGGAAGAGAGAGAGGAGGTG
	p53	GGAAATTTGAGTATGGAGTATTT	GTTGTAGTGGATGGTGGTACAG
	Gapdh	GGTGTGAACCATGAGAAGTATGA	GAGTCCTTCCACGATACCAAAG

Supplementary Table S3. The human primer sequences used for ChIP analysis in this study

Genomic location	Forward primers (5′-3′)	Reverse primers (3′-5′):
p53 -5 kb	GAGACGGAGTCTTACACTGTTG	GAGTCTGAGGCAGGAGAATTAC
p53 -3 kb	GCACAAAGCTATTTCCCAGTTC	GAGGATAAGGGATCAGTCATGTG
p53 -1.5 kb	CAGAGGTATCTTCCATGGCTTT	CGAGGAGCAAGAACTTTCTGA
p53 -1 kb	CCGACGCAGAGCTAAAGAAA	AATTGGCGTCCGCTGTT
p53 -0.5 kb	TCAGACCTGTCTCCCTCATT	TGTTGTATTCCTGAGTGCCTATATC
p53 -0.3 kb	AGGGTGTGATATTACGGAAAGC	ATGAAGGGTGGAAGGAAGAAAG
p53 -0.1 kb	AATGTTAGTATCTACGGCACCAG	CCATGACAAGTAAGGGCAAGTA
p53 TSS	GCTCAAGACTGGCGCTAAA	GTGTCACCGTCGTGGAAAG
p53 +0.1 kb	GCTTCCCTGGATTGGGTAA	AAATACACGGAGCCGAGAG
p53 +0.5 kb	GCAGAGGAGTGGAGCTTTG	CCTCAATGCTTTGTGCATCTTT
p53 +3 kb	CAGTAAGGTCCTTGAGCCATTA	ACTGGATTTCCATACAGGAAAGA
p53 +11 kb	CAGGGTTGGAAGTGTCTCAT	TCGACGCTAGGATCTGACT

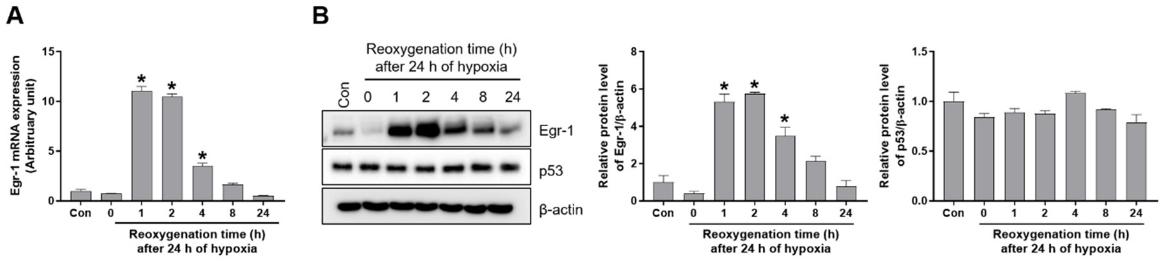

Supplementary Figure S1. Egr-1 and p53 expression is induced by hypoxia and reoxygenation in renal distal tubular cells. Distal tubular MDCT cells were exposed to 24 h of hypoxia followed by reoxygenation at the indicated times. (A) Egr-1 mRNA expression was measured by real-time quantitative PCR analysis (B) The protein expression of Egr-1 and p53 was measured by Western blot analysis; relative protein levels were determined with β-actin as a loading control. The values are expressed as mean ± SEM. *P < 0.05 vs. control group.
